# Reply: Thymidylate synthase polymorphism and survival of colorectal cancer patients treated with 5-fluorouracil

**DOI:** 10.1038/sj.bjc.6600230

**Published:** 2002-04-22

**Authors:** B Iacopetta, H Elsaleh

**Affiliations:** School of Surgery and Pathology, University of Western Australia, Crawley, 6009 WA, Australia

## Abstract

*British Journal of Cancer* (2002) **86**, 1366. DOI: 10.1038/sj/bjc/6600230 www.bjcancer.com

© 2002 Cancer Research UK

## Sir

Drs Ulrich and Potter appear to have missed the point of our study on the predictive value of the polymorphism in the thymidylate synthase (TS) promoter (Iacopetta *et al*, 2001). The aim was to determine whether this genotype was associated with the degree of survival benefit gained from the use of adjuvant, 5-fluorouracil-based (5FU) chemotherapy in colorectal cancer patients. To achieve this aim, we compared the survival of patients treated by surgery alone to those treated by surgery and chemotherapy. There was a clear long-term survival benefit for the combined 2R/2R and 2R/3R patient group, but not for the 3R/3R group. We agree however that the predictive value of the 3R/3R genotype is weak and, as stated in the Discussion of our article direct biochemical or immunohistochemical evaluation of TS level is likely to be required for clinical applications relating to patient selection for 5FU chemotherapy. Investigation of the prognostic value of TS genotypes within the chemotherapy group, as suggested by Ulrich and Potter, was not our primary aim. As shown in the Results of our article, the survival of 3R/3R patients in this group was marginally worse than that of 2R/2R or 2R/3R patients, presumably because they gained less survival benefit from the adjuvant treatment.

It is interesting to note that during the publication of our manuscript there have been two other reports on the predictive value of TS genotype. The first by Villafranca *et al* (2001) showed that rectal tumours from patients with the 3R/3R genotype showed a lower probability of down-staging after pre-operative chemo-(5FU)-radiation treatment compared to 2R/2R or 2R/3R genotypes (22% *vs* 60%, respectively). The second by Pullarkat *et al* (2001) showed that metastatic colorectal cancer patients with the 3R/3R genotype had a lower response rate (9%) to treatment with 5FU compared to those with the 2R/2R genotype (50%). Both findings are in agreement with our observations of less survival benefit from 5FU treatment for 3R/3R genotype patients compared to those with 2R/2R or 2R/3R genotypes.
